# Study on the Clinical Features of Parkinson's Disease With Probable Rapid Eye Movement Sleep Behavior Disorder

**DOI:** 10.3389/fneur.2020.00979

**Published:** 2020-09-11

**Authors:** Kexin Long, Changmin Wan, Yaqin Xiang, Jiabin Liu, Qian Xu, Qiying Sun, Zhiqin Wang, Yun Tian, Liangjuan Fang, Yang Yang, Xinxiang Yan, Beisha Tang, Jifeng Guo

**Affiliations:** ^1^Department of Neurology, Xiangya Hospital, Central South University, Changsha, China; ^2^Department of Neurology, Changsha Central Hospital, Changsha, China; ^3^National Clinical Research Center for Geriatric Disorders, Changsha, China; ^4^Key Laboratory of Hunan Province in Neurodegenerative Disorders, Central South University, Changsha, China

**Keywords:** Parkinson's disease, sleep disorders, probable rapid eye movement sleep behavior disorder, motor symptoms, non-motor symptoms

## Abstract

**Objective:** To investigate the clinical features and factors associated with Parkinson's disease (PD) patients with probable rapid eye movement sleep behavior disorder (PD-pRBD).

**Methods:** A total of 2,440 patients with clinically established or clinically probable PD were divided into two groups: PD-pRBD and PD without pRBD (PD-NRBD), according to the RBD questionnaire—Hong Kong. Data collection included demographic data, basic clinical history, and motor and non-motor symptoms. Based on the onset time of pRBD and the motor symptoms in PD, PD-pRBD patients were further divided into the pRBD prior to PD (PD-prRBD) group and the pRBD posterior to PD (PD-poRBD) group. Clinical features were compared between the PD-pRBD and PD-NRBD groups, as well as the PD-prRBD and PD-poRBD groups. The associated factors of pRBD were also explored.

**Results:** The prevalence of pRBD was 41.4% (1,010 out of the total of 2,440) in our PD cohort. Further, compared with the PD-NRBD group, the PD-pRBD group had longer disease duration and more severe motor symptoms. Moreover, the PD-pRBD group had significantly higher levodopa equivalent daily dose and a higher ratio of dyskinesia, wearing-off, and offset of the Hoehn–Yahr stage. The scores on the non-motor symptom rating scale (NMSS), cognitive impairment, Parkinson's disease sleep scale (PDSS), excessive daytime sleepiness, constipation, hyposmia, depression, and the 39-item Parkinson's disease questionnaire also appeared worse in the PD-pRBD group. Significant differences in the educational level, disease duration, disease progression, Unified Parkinson's Disease Rating Scale (UPDRS)-II, UPDRS-III, tremor, rigidity, bradykinesia, posture gait, frozen gait, levodopa equivalent daily dose, dyskinesia, wearing-off, Hoehn–Yahr stage, NMSS-6, PDSS, and communication score widely existed between the PD-prRBD and PD-poRBD groups. Late-onset PD, long disease duration, high UPDRS-I score, high NMSS-4 score, low PDSS score, constipation, and hyposmia were all identified as the risk factors for PD-pRBD.

**Conclusions:** Compared with the PD-NRBD group, the PD-pRBD group may have more severe motor symptoms, motor complications, and non-motor symptoms as well as a substandard quality of life. Further, late-onset PD, long disease duration, high UPDRS-I score, high NMSS-4 score, low PDSS score, constipation, and hyposmia can be risk factors for RBD in PD. Differences also occurred between the PD-prRBD and PD-poRBD groups.

## Introduction

Rapid eye movement sleep behavior disorder (RBD) is a common sleep disorder in patients with Parkinson's disease (PD). It is characterized by violent behaviors with dreams during nighttime sleep and often causes harm to the patients and their bed partners. RBD can occur several decades before the occurrence of PD and is currently considered as a precursor of PD. Patients with primary RBD have a higher risk of developing neurodegenerative diseases (1–4). Moreover, PD patients with RBD experience more severe motor symptoms, autonomic symptoms, and cognitive dysfunction than PD patients without RBD (5). Amid the development of disease modification therapy, the identification of prodromal symptoms of PD, such as RBD, olfactory dysfunctions, and depression, are essential for early diagnosis and treatment of the disease. Recently, the study of RBD in PD has become a hot topic. However, few large-sample studies have focused on the clinical features and risk factors of RBD in Chinese patients. There are still some issues that remain uncertain and require further research. Our study recruited PD patients to explore the clinical features and related factors of RBD.

## Materials and Methods

### Subjects

All the PD patients were recruited from the inpatients and outpatients of the Department of Neurology of Xiangya Hospital, Central South University, Hunan, China, between February 2017 and April 2018 at Parkinson's Disease & Movement Disorders Multicenter Database and Collaborative Network in China (PDMDCNC, http://pd-mdcnc.com:3111/). The subjects were diagnosed by a neurological specialist, according to the 2015 International Parkinson and Movement Disorder Society Clinical Diagnostic Criteria for PD (6). Patients who met the following criteria were excluded: (1) patients with speech impairment or other reasons leading to incomplete data collection; (2) malignant tumors or other serious systemic diseases; and (3) RBD secondary to diseases other than PD. The Ethics Committee of Xiangya Hospital of Central South University approved this study, and all patients provided written informed consent.

### Assessments

Body mass index (BMI) was calculated as height/body weight^2^ (meters per kilogram squared). PD patients were divided into two groups according to the onset of PD motor symptoms: early-onset Parkinson's disease (EOPD) and late-onset Parkinson's disease (LOPD). The age of onset of EOPD patients was ≤50 years old, and the age of onset of the LOPD patient was >50 years.

The patients' motor symptoms were assessed according to the Unified Parkinson's Disease Rating Scale (UPDRS)-II, UPDRS-III tremor scores, and posture gait score. Patients with PD can be divided into three motor subtypes: tremor-dominant PD, posture instability and gait difficulty PD (PIGD PD), and mixed PD (7, 8). The total tremor scores, including UPDRS II-16, UPDRS III-20, and UPDRS III-21, divided by eight, is the average tremor score. The total posture and gait scores, including UPDRS II-13, UPDRS II-14, UPDRS II-15, UPDRS III-29, and UPDRS III-30, divided by five, is the average score of posture gait. The average tremor score/posture gait average score ≥1.5 is defined as tremor-dominant PD; the tremor average score/posture gait average score ≤1.0 is defined as PIGD PD, whereas 1.0 < average tremor score/posture gait average score <1.5 is defined as mixed PD (8). The patient's medication was expressed by levodopa equivalent daily dose (LEDD) (9). Additionally, the Hoehn–Yahr (H-Y) stage was used to assess the severity of PD. According to the H-Y stage, PD patients can be classified into early PD group (H-Y stage 1–2), medium group (H-Y stage 2.5–3), and late PD group (H-Y stage 4–5).

RBD was assessed using the rapid eye movement sleep behavior disorder questionnaire-Hong Kong (RBDQ-HK). The best cutoff values of RBDQ-HK were 18 points on the RBDQ-HK overall scale with a sensitivity of 86.9% and specificity of 70.6% in PD patients (10). PD patients with RBDQ-HK ≥ 18 points were categorized into the PD patients with probable rapid eye movement sleep behavior disorder (PD-pRBD) group, whereas patients with RBDQ-HK <18 points were categorized into the PD without pRBD (PD-NRBD) group. PD-pRBD patients with recorded pRBD onset time were divided into two groups based on the order of the onset time of pRBD and the motor symptoms in PD: pRBD prior to PD (PD-prRBD) and posterior to PD (PD-poRBD).

The non-motor symptom rating scale (NMSS) was used for the evaluation of non-motor symptoms in PD patients. There were 30 questions covering nine aspects with the frequency and severity of each symptom; the higher the score, the more severe the symptoms (11). The Parkinson's disease sleep scale (PDSS) was used to evaluate the quality of sleep in patients with PD (12). The Epworth Sleepiness Scale was used to screen patients with PD for excessive daytime sleepiness (EDS). Epworth Sleepiness Scale total score > 10 can be diagnosed with EDS (13).

The cognitive function of PD patients was assessed using the Mini-Mental State Examination (MMSE). The MMSE contains 30 questions and five aspects: orientation, registration, attention and calculation power, recall, and language. The threshold of cognitive dysfunction is related to the education level and is as follows—illiterate group: ≤17 points, primary school group: ≤20 points, and middle school or above group: ≤24 points; values above the threshold indicates the normal cognitive function, whereas values below the threshold indicate cognitive dysfunction (14).

The Hamilton Depression Scale-17 item version was used to assess whether patients with PD had a depressed state (15). A Hamilton Depression Scale-17 item version score of up to seven points indicates there is no depression; a score > 7 points indicates depression (16).

Patients with PD were assessed for constipation using the Functional Constipation Rome III diagnostic criteria.

The Hyposmia Rating Scale was used to screen subjects for an olfactory disturbance. If Hyposmia Rating Scale is no more than 22.5 points, there is an olfactory sensation (17).

The 39-item Parkinson's Disease Questionnaire (PDQ-39) consists of 39 questions and eight aspects that evaluate the PD patients' quality of life. The eight aspects include mobility, activities of daily living, emotional well-being, stigma, social support, cognition, communication, and bodily discomfort. The higher the score, the worse the quality of life of the subjects (18).

### Statistical Analysis

Continuous variables are expressed as “mean ± standard deviation,” and the *t*-test was used to compare the clinical features between PD-pRBD and PD-NRBD, as well as the clinical features between PD-prRBD and PD-poRBD. The statistical differences in the categorical variables were calculated using the chi-square test. Multivariate logistic regression analysis was used to evaluate the factors associated with RBD. Data processing and analysis were performed using SPSS 20.0. A *P*-value < 0.05 was considered statistically significant.

## Results

A total of 2,440 PD patients were eventually included, whereas 59 patients were excluded because of incomplete data. Further, 1,010 PD patients (41.4% of 2,440) were categorized into the PD-pRBD group, whereas 1,430 PD patients (58.6% of 2,440) were categorized into the PD-NRBD group. Among PD-pRBD patients, the PD-prRBD group comprised 348 patients and the pPD-poRBD group comprised 544 patients.

### Comparison of Clinical Features Between the PD-pRBD and PD-NRBD Groups

#### Comparison of Demographic Data

The mean ages of the PD-pRBD group and the PD-NRBD group were 64.79 ± 9.10 and 60.64 ± 10.10 years, respectively, indicating that the patients in the PD-pRBD group were significantly older. In terms of education, the PD-pRBD group had a significantly higher proportion of patients with primary education and below (36.1% of PD-pRBD group vs. 28.9% of PD-NRBD). The mean BMI between the PD-pRBD and PD-NRBD groups was significantly different. There was no significant difference in sex ratio between the PD-pRBD and PD-NRBD groups. These findings are shown in Table 1.

**Table 1:** Comparison of demographic data between PD-pRBD and PD-NRBD.

**Variable**	**PD-pRBD**	**PD-NRBD**	***P***
	**(*n* = 1,010)**	**(*n* = 1,430)**	
Age (years)	64.79 ± 9.10	60.64 ± 10.10	<0.001^*^
Sex (male/female, *n*)	513/497	739/691	0.666
Educational level^a^	365/536/109	412/816/202	<0.001^*^
BMI (kg/m^2^)	22.42 ± 3.18	22.83 ± 3.02	0.002^*^

a *Primary school and below/secondary school/university and above, cases; BMI, body mass index*.

* *Statistically significant (p < 0.05)*.

#### Comparison of Lifestyle and Environmental Factors

As shown in Table 2, no significant differences were observed in smoking, alcohol consumption, tea-drinking, and pesticide exposure between the PD-pRBD and PD-NRBD groups. It seems that the earlier mentioned lifestyle and environmental factors did not affect the occurrence of PD-pRBD.

**Table 2:** Comparison of lifestyle and environmental factors between PD-pRBD and PD-NRBD.

**Variable**	**PD-pRBD**	**PD-NRBD**	***P***
	**(*n* = 1,010)**	**(*n* = 1,430)**	
Smoking (yes/no, *n*)	231/779	296/1,134	0.199
Alcohol (yes/no, *n*)	195/815	271/1,159	0.826
Tea drinking (yes/no, *n*)	115/895	168/1,262	0.783
Pesticide exposure (yes/no, *n*)	164/846	222/1,208	0.634

#### Comparison of Age at Onset and Motor Symptoms

Table 3 presents the detailed results regarding the age of onset and motor symptoms. First, the PD-pRBD group had a higher LOPD ratio. There were 232 (23.0%) cases of EOPD and 778 (77.0%) cases of LOPD in the PD-pRBD group; there were 481 (33.6%) cases of EOPD and 949 (66.4%) cases of LOPD in the PD-NRBD group. However, no significant difference was observed in the initial presentation of motor symptoms.

**Table 3:** Comparison of age of onset and motor symptoms between PD-pRBD and PD-NRBD.

**Variable**	**PD-pRBD**	**PD-NRBD**	***P***
	**(*n* = 1,010)**	**(*n* = 1,430)**	
EOPD/LOPD (*n*)	232/778	481/949	<0.001^*^
Initial presentation of	400/409/192/9	593/564/263/10	0.784
motor symptoms^a^			
UPDRS-I score	3.17 ± 2.02	2.13 ± 1.87	<0.001^*^
UPDRS-II score	13.83 ± 6.62	11.23 ± 5.96	<0.001^*^
UPDRS-III score	29.76 ± 14.44	26.02 ± 14.16	<0.001^*^
Tremor score	3.42 ± 3.45	3.30 ± 3.38	0.406
Rigidity score	5.98 ± 4.17	5.34 ± 3.93	<0.001^*^
Bradykinesia score	11.19 ± 6.31	9.88 ± 6.16	<0.001^*^
Posture gait score	5.05 ± 3.11	4.00 ± 2.93	<0.001^*^
Motor subtype^b^	109/786/115	225/1,021/184	<0.001^*^
Frozen gait (with/without, *n*)	304/706	260/1,170	<0.001^*^
LEDD (mg)	411.89 ± 314.21	343.37 ± 299.38	<0.001^*^
Dyskinesia (with/without, *n*)	183/827	163/1,267	<0.001^*^
Wearing-off (with/without, *n*)	253/757	235/1,195	<0.001^*^
H-Ystage^c^	398/528/84	791/569/70	<0.001^*^
Disease duration (years)	6.89 ± 5.12	5.20 ± 4.16	<0.001^*^
Disease progression (score/years)	7.10 ± 7.09	8.07 ± 7.46	0.001^*^

a *Tremor/bradykinesia/tremor + bradykinesia/others, cases*.

b *TD PD/PIGD PD/M PD, cases*.

c *Stage 1–2/stage 2.5–3/stage 4–5, cases*.

*EOPD, early-onset Parkinson's disease; LOPD, late-onset Parkinson's disease; UPDRS, Unified Parkinson's Disease Rating Scale; PIGD, postural instability/gait disorder; TD, tremor dominant; LEDD, levodopa equivalent dose daily*.

* *Statistically significant (p < 0.05)*.

Regarding motor symptoms, the PD-pRBD group had higher scores for UPDRS-I, UPDRS-II, and UPDRS-III. The PD-pRBD group had significantly higher rigidity, bradykinesia, and posture gait scores. No significant difference was found in the tremor score between the two groups. In the PD-pRBD group, there were 304 patients (30.1%) with frozen gait, whereas only 260 patients (18.2%) had frozen gait in the PD-NRBD group. The PD-pRBD group had a significantly higher proportion of patients with frozen gait.

Regarding the motor subtypes, there were significant differences in motor subtype; the PD-pRBD group had a higher proportion of patients with PIGD PD.

Moreover, patients in the PD-pRBD group had a longer disease duration. The PD-pRBD group patients also had a significantly slower disease progression than the PD-NRBD group. The H-Y staging composition was also completely different between the two groups: the PD-pRBD group was more likely to stay in the middle and late stages of PD.

**Figure 1:**
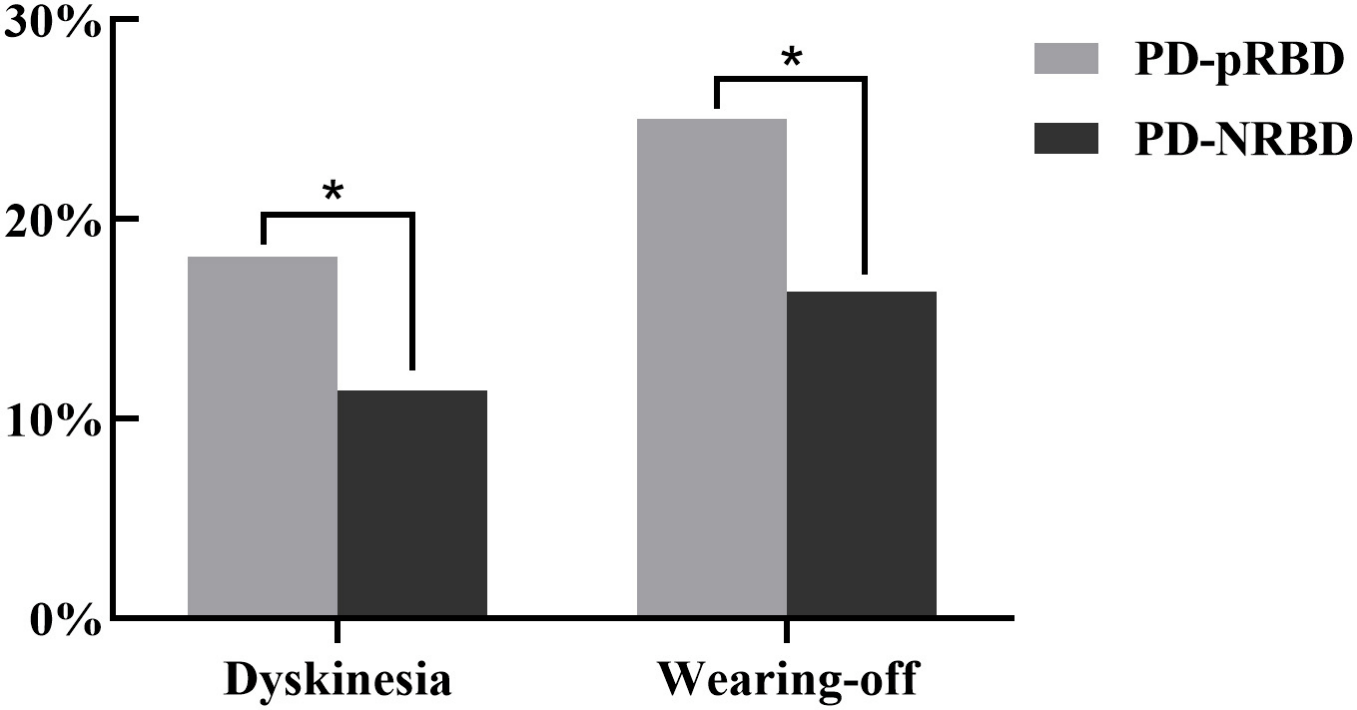
Comparison of motor complications between PD-pRBD and PD-NRBD (*p < 0.05).

Furthermore, LED was significantly different between the two groups. Patients in the PD-pRBD group had a higher ratio of dyskinesia and wearing-off. A comparison of motor complications between PD-pRBD and PD-NRBD is shown in Figure 1.

#### Comparison of Non-motor Symptoms

**Figure 2:**
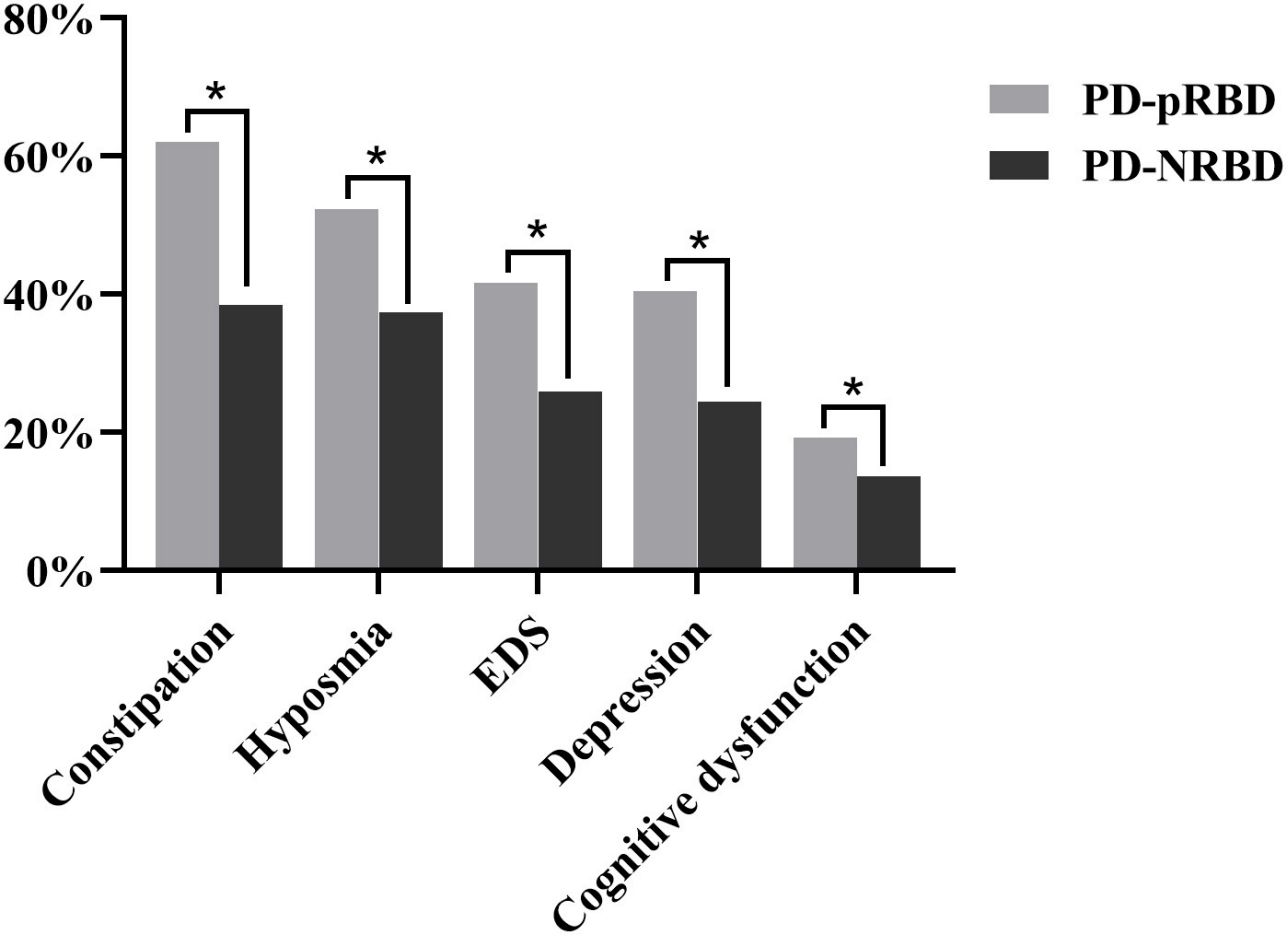
Comparison of non-motor symptoms between PD-pRBD and PD-NRBD (*p < 0.05).

In addition to the motor symptoms, the PD-pRBD group patients also exhibited worse non-motor symptoms. The findings results are shown in Table 4 and Figure 2. The scores of items 1–7 and 9 in the NMSS were all higher in the PD-pRBD group, which indicates that non-motor symptoms such as orthostatic hypotension, urinary system symptoms, and gastrointestinal symptoms were more severe in this group.

**Table 4:** Comparison of non-motor symptoms between PD-pRBD and PD-NRBD.

**Variable**	**PD-pRBD**	**PD-NRBD**	***P***
	**(*n* = 1,010)**	**(*n* = 1,430)**	
NMSS-1 score	1.31 ± 2.25	0.78 ± 1.70	<0.001^*^
NMSS-2 score	10.70 ± 7.33	7.83 ± 6.65	<0.001^*^
NMSS-3 score	8.40 ± 9.86	6.03 ± 8.62	<0.001^*^
NMSS-4 score	1.68 ± 3.24	0.68 ± 1.77	<0.001^*^
NMSS-5 score	4.56 ± 4.40	3.09 ± 3.67	<0.001^*^
NMSS-6 score	5.82 ± 4.81	3.50 ± 4.22	<0.001^*^
NMSS-7 score	7.22 ± 6.50	5.16 ± 5.82	<0.001^*^
NMSS-9 score	6.10 ± 5.00	4.79 ± 4.73	<0.001^*^
Cognitive dysfunction (with/without, *n*)	194/816	195/1,235	<0.001^*^
Orientation score	9.29 ± 1.32	9.50 ± 1.07	<0.001^*^
Registration score	2.74 ± 0.59	2.79 ± 0.52	0.022^*^
Attention and calculation score	3.56 ± 1.52	3.86 ± 1.44	<0.001^*^
Recall score	2.06 ± 1.04	2.14 ± 0.98	0.044^*^
Language score	7.96 ± 1.38	8.22 ± 1.19	<0.001^*^
PDSS score	107.40 ± 25.93	120.84 ± 23.67	<0.001^*^
EDS (with/without, *n*)	421/589	370/1,060	<0.001^*^
Constipation (with/without, *n*)	626/384	550/880	<0.001^*^
Hyposmia (with/without, *n*)	528/482	535/895	<0.001^*^
Depression (with/without, *n*)	409/601	349/1081	<0.001^*^

*NMSS, non-motor symptom rating scale; PDSS, Parkinson's Disease Sleep Scale; EDS, excessive daytime sleepiness*.

* *Statistically significant (p < 0.05)*.

Moreover, when considering cognitive dysfunction, the PD-pRBD group patients were also in worse condition. There were 194 (19.2%) patients in the PD-pRBD group and 195 (13.6%) patients in the PD-NRBD group. Specifically, the average scores of orientation, registration, attention and calculation, recall, and language ability were all significantly lower in the PD-pRBD group. This indicates that RBD may have a comprehensive effect on cognitive function.

Furthermore, patients in the PD-pRBD group had lower total PDSS scores and more EDS.

We further demonstrated that RBD is associated with constipation, hyposmia, and depression. There were significant differences in all three symptoms between the PD-pRBD group and the PD-NRBD group. In these three items, the PD-pRBD group behaved worse.

#### Comparison of Quality of Life

The PDQ-39 score was significantly different between the PD-pRBD and PD-NRBD groups. The PD-pRBD group had higher PDQ-39 scores, and the quality of life was worse. In particular, statistically significant differences in mobility, activities of daily living, emotional well-being, social support, cognition, communication, and bodily discomfort were observed between the two groups. However, the condition of stigma remained the same. These results are shown in Table 5.

**Table 5:** Comparison of quality of life between PD-pRBD and PD-NRBD.

**Variable**	**PD-pRBD**	**PD-NRBD**	***P***
	**(*n* = 1,010)**	**(*n* = 1,010)**	
PDQ-39 score	37.60 ± 25.78	25.62 ± 23.49	<0.001^*^
Mobility score	11.51 ± 11.32	7.29 ± 9.85	<0.001^*^
Activities of daily	6.90 ± 6.35	4.77 ± 5.76	<0.001^*^
living score			
Emotional well-being score	5.78 ± 6.02	4.18 ± 5.38	<0.001^*^
Stigma score	3.65 ± 4.75	3.39 ± 4.57	0.170
Social support score	0.48 ± 1.44	0.33 ± 1.31	<0.001^*^
Cognitions score	5.08 ± 3.23	2.60 ± 2.63	<0.001^*^
Communication score	1.55 ± 2.31	1.10 ± 1.96	<0.001^*^
Bodily discomfort score	2.64 ± 2.58	1.91 ±2.28	<0.001^*^

*PDQ-39, 39-item Parkinson's Disease Questionnaire*.

* *Statistically significant (p < 0.05)*.

### Binary Logistic Regression Analysis of Risk Factors for Parkinson's Disease Patients With Probable Rapid Eye Movement Sleep Behavior Disorder

The earlier mentioned statistically significant factors, except for repeated components, were included in the binary logistic regression model to investigate the risk factors of PD-pRBD. The results revealed that LOPD, long disease duration, high UPDRS-I score, high NMSS-4 score, low total PDSS score, constipation, and hyposmia were risk factors for PD-pRBD. The results of these analyses are presented in Table 6.

**Table 6:** Logistic regression analysis of risk factors for PD-pRBD.

**Variable**	***P***	**Exp(B)**	**OR (95% CI)**
LOPD	<0.001^*^	1.861	1.429, 2.425
Disease duration	<0.001^*^	1.062	1.033, 1.091
UPDRS-I score	<0.001^*^	1.182	1.110, 1.258
NMSS-4 score	0.003^*^	1.085	1.029, 1.145
PDSS score	<0.001^*^	0.989	0.984, 0.993
Constipation	<0.001^*^	1.541	1.260, 1.884
Hyposmia	<0.001^*^	1.698	1.353, 2.133

*LOPD, late-onset Parkinson's disease; UPDRS, Unified Parkinson's Disease Rating Scale; NMSS, non-motor symptom rating scale; PDSS, Parkinson's Disease Sleep Scale*.

* *Statistically significant (p < 0.05)*.

### Comparison of Clinical Features Between the PD-prRBD and PD-poRBD Groups

#### Comparison of Demographic Data

Among the 1,010 PD-pRBD patients, 892 patients could recall the onset time of pRBD or the relationship between the onset time of pRBD and PD motor symptoms, including 348 patients with PD-prRBD and 544 patients with PD-poRBD. When comparing the PD-prRBD and PD-poRBD groups, only the educational level showed a significant difference. The PD-prRBD group had a higher proportion of patients with primary education and below than the PD-poRBD group. These results are shown in Table 7.

**Table 7:** Comparison of demographic data between PD-prRBD and PD-poRBD.

**Variable**	**PD-prRBD**	**PD-poRBD**	***P***
	**(*n* = 348)**	**(*n* = 544)**	
Age (years)	65.45 ± 8.94	64.29 ± 8.97	0.060
Sex (male/female, *n*)	171/177	277/267	0.604
Educational level^a^	144/173/31	176/307/61	0.021^*^
BMI (kg/m^2^)	22.71 ± 3.11	22.26 ± 3.19	0.054

a *Primary school and below/secondary school/university and above, cases*.

*BMI, body mass index*.

* *Statistically significant (p < 0.05)*.

#### Comparison of Lifestyle and Environmental Factors

There were no significant differences in smoking, alcohol consumption, tea-drinking, and pesticide exposure between the PD-prRBD and PD-poRBD groups. These results are shown in Table 8.

**Table 8:** Comparison of lifestyle and environmental factors between PD-prRBD and PD-poRBD.

**Variable**	**PD-prRBD**	**PD-poRBD**	***P***
	**(*n* = 348)**	**(*n* = 544)**	
Smoking (yes/no, *n*)	80/268	126/418	0.952
Alcohol (yes/no, *n*)	76/272	95/449	0.105
Tea drinking (yes/no, *n*)	39/309	63/481	0.864
Pesticide exposure (yes/no, *n*)	64/284	77/467	0.091

#### Comparison of Age at Onset and Motor Symptoms

**Figure 3:**
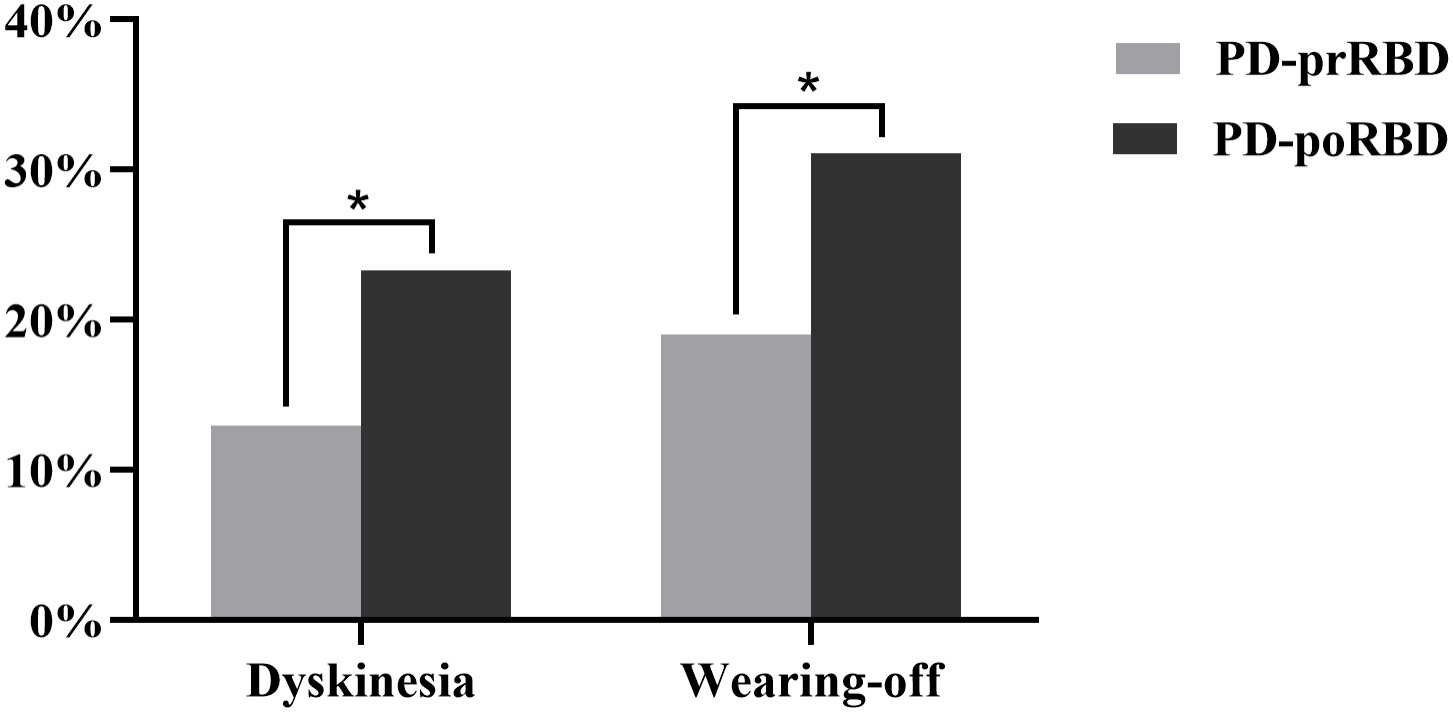
Comparison of motor complications between PD-prRBD and PD-poRBD (*p < 0.05).

Table 9 and Figure 3 summarize the age of onset and motor symptoms of PD-prRBD and PD-poRBD. The PD-prRBD group had a higher LOPD ratio. Moreover, the PD-poRBD group had higher scores for UPDRS-II, UPDRS-III, tremor, rigidity, bradykinesia, and posture gait. Also, the PD-poRBD group had a higher proportion of patients with frozen gait. We found that the PD-poRBD group had a longer disease duration and slower disease progression than those in the PD-prRBD group. Concerning the H-Y staging, the PD-poRBD group had a higher proportion of patients in the middle and late stages of PD. The LEDD required by the PD-poRBD group was significantly larger. Meanwhile, the incidence of dyskinesia and wearing-off was also higher in the PD-poRBD group.

**Table 9:** Comparison of age of onset and motor symptoms between PD-prRBD and PD-poRBD.

**Variable**	**PD-prRBD**	**PD-poRBD**	***P***
	**(*n* = 348)**	**(*n* = 544)**	
EOPD/LOPD (*n*)	51/297	156/388	<0.001^*^
Initial presentation of	143/147/58/0	211/217/107/9	0.061
motor symptoms^a^			
UPDRS-I score	3.20 ± 1.99	3.15 ± 2/03	0.741
UPDRS-II score	12.45 ± 6.23	14.88 ± 6.48	<0.001^*^
UPDRS-III score	27.86 ± 13.17	31.45 ± 15.01	<0.001^*^
Tremor score	3.11 ± 3.01	3.60 ± 3.71	0.028^*^
Rigidity score	5.68 ± 3.83	6.40 ± 4.29	0.009^*^
Bradykinesia score	10.63 ± 6.03	11.67 ± 6.53	0.017^*^
Posture gait score	4.54 ± 2.81	5.39 ± 3.19	<0.001^*^
Motor subtype^b^	48/257/43	58/420/66	0.354
Frozen gait (with/without, *n*)	84/264	196/348	<0.001^*^
LEDD (mg)	349.19 ± 314.63	468.88 ± 305.14	<0.001^*^
Dyskinesia (with/without, *n*)	45/303	126/418	<0.001^*^
Wearing-off (with/without, *n*)	66/282	169/375	<0.001^*^
H-Ystage^c^	161/166/21	189/303/52	0.002^*^
Disease duration (years)	5.09 ± 3.91	8.26 ± 5.30	<0.001^*^
Disease progression (score /years)	9.28 ± 8.59	5.41 ± 5.26	<0.001^*^

a *Tremor/bradykinesia/tremor + bradykinesia/others, cases*.

b *TD PD/PIGD PD/M PD, cases*.

c *Stage 1–2/stage 2.5–3/stage 4–5, cases*.

*EOPD, early-onset Parkinson's disease; LOPD, late-onset Parkinson's disease; UPDRS, Unified Parkinson's Disease Rating Scale; PIGD, postural instability/gait disorder; TD, tremor dominant; LEDD, levodopa equivalent dose daily*.

* *Statistically significant (p < 0.05)*.

#### Comparison of Non-motor Symptoms

Considering the non-motor symptoms, the PD-poRBD group had higher NMSS-6 scores and lower PDSS scores. These significant differences indicated that the PD-poRBD group had more severe gastrointestinal symptoms and worse sleep quality. However, no significant difference was found in other non-motor evaluations. These results are described in detail in Table 10.

**Table 10:** Comparison of non-motor symptoms between PD-prRBD and PD-poRBD.

**Variable**	**PD-prRBD**	**PD-poRBD**	***P***
	**(*n* = 348)**	**(*n* = 544)**	
NMSS-1 score	1.29 ± 2.18	1.29 ± 2.24	0.986
NMSS-2 score	10.36 ± 7.04	11.14 ± 7.43	0.121
NMSS-3 score	8.36 ± 9.56	8.65 ± 10.28	0.680
NMSS-4 score	1.64 ± 3.01	1.69 ± 3.34	0.817
NMSS-5 score	4.60 ± 4.21	4.67 ± 4.61	0.815
NMSS-6 score	5.30 ± 4.68	6.27 ± 4.92	0.003^*^
NMSS-7 score	7.04 ± 6.42	7.46 ± 6.67	0.354
NMSS-9 score	5.75 ± 4.86	6.18 ± 4.87	0.194
Cognitive dysfunction	59/289	107/437	0.309
(with/without, *n*)			
Orientation score	9.28 ± 1.23	9.34 ± 1.35	0.520
Registration score	2.74 ± 0.58	2.74 ± 0.59	0.934
Attention and calculation score	3.49 ± 1.53	3.60 ± 1.51	0.306
Recall score	2.04 ± 1.06	2.11 ± 0.99	0.272
Language score	7.94 ± 1.35	7.98 ± 1.43	0.691
PDSS score	110.57 ± 24.92	105.53 ± 26.02	0.004^*^
EDS (with/without, *n*)	150/198	226/318	0.645
Constipation (with/without, *n*)	206/142	350/194	0.122
Hyposmia (with/without, *n*)	174/174	300/244	0.133
Depression (with/without, *n*)	126/222	230/314	0.071

*NMSS, non-motor symptom rating scale; PDSS, Parkinson's Disease Sleep Scale; EDS, excessive daytime sleepiness*.

* *Statistically significant (p < 0.05)*.

#### Comparison of Quality of Life

There were no differences in the total scores of PDQ-39 between the two groups; however, the average communication score showed a slight difference. These results are shown in Table 11.

**Table 11:** Comparison of quality of life between PD-prRBD and PD-poRBD.

**Variable**	**PD-prRBD**	**PD-poRBD**	***P***
	**(*n* = 348)**	**(*n* = 544)**	
PDQ-39 score	35.10 ± 26.09	40.32 ± 25.46	0.143
Mobility score	10.33 ± 11.56	12.54 ± 11.12	0.299
Activities of daily living score	6.06 ± 6.10	7.68 ± 6.37	0.317
Emotional well-being score	5.56 ± 5.95	6.03 ± 6.15	0.059
Stigma score	3.32 ± 4.55	3.96 ± 4.94	0.572
Social support score	0.49 ± 1.56	0.50 ± 1.41	0.902
Cognitions score	5.30 ± 3.26	5.12 ± 3.23	0.065
Communication score	1.33 ± 2.15	1.75 ± 2.40	0.047^*^
Bodily discomfort score	2.61 ± 2.58	2.73 ± 2.62	0.470

*PDQ-39, 39-item Parkinson's Disease Questionnaire*.

* *Statistically significant (p < 0.05)*.

## Discussion

PD is a common neurodegenerative disease among the population over the age of 60 years. It is believed that patients with PD have a series of non-motor symptoms before the onset of motor symptoms. Initially, degeneration of the olfactory bulb and intestinal wall plexus cells in patients with PD can lead to olfactory or constipation symptoms. Damage to the medulla oblongata and pons can cause sleep disorders and depression. With the progress of the disease, the dopaminergic neurons in the substantia nigra pars compacta are damaged, which directly leads to the motor symptoms of PD. Finally, cognitive dysfunction gradually appears when the lesion involves the cerebral cortex (19).

In PD patients, RBD is the most common sleep disorder in addition to insomnia. The prevalence of RBD in patients with PD is 39–46%, and the prevalence of RBD in early-onset PD patients is approximately 30% (20). However, the reported incidence of RBD varies widely because of the differences in sample sizes, assessment methods, and diagnostic criteria for sleep disorders. Previous studies revealed that 28–82% of primary RBD patients could develop into PD, multiple system atrophy, Lewy body dementia, and other neurological α-synuclein diseases (20–23). In a longitudinal cohort study consisting of 174 patients with primary RBD, 17.7, 40.6, and 52.4% of patients developed neurodegenerative diseases, including PD in the 5th, 10th, and 12th years, respectively (24). Secondary RBD can occur early after PD onset. In a study of 231 patients with PD, ~30% had RBD symptoms within 4 years after PD onset. Growing evidence has shown that PD patients with pRBD are more severe in both motor and non-motor symptoms than those without pRBD (25).

In this study, we have reported the differences in general information, motor symptoms, non-motor symptoms, motor complications, and quality of life between PD-pRBD and PD-NRBD, as well as between PD-prRBD and PD-poRBD. These data have provided several novel observations and a more comprehensive comparison among these groups.

In this study, we found that LOPD, long disease duration, high UPDRS-I score, high NMSS-4 score, low PDSS score, constipation, and hyposmia could be risk factors for PD-pRBD. It has been previously shown that idiopathic RBD patients combined with olfactory dysfunction are more likely to develop neurodegenerative diseases, such as PD, in a relatively short time (26). Early identification and treatment of pRBD and pRBD related risk factors help delay disease progression and improve patient quality of life.

In contrast to previous studies (27, 28), our study found no significant difference in sex between the PD-pRBD and PD-NRBD groups. The discrepancy in findings might be due to an underestimation of the prevalence of pRBD in female patients. Further, male patients often experience anger, fear, and violence in their dreams, whereas women experience fear-based emotions (29); more RBD of women could be ignored. We also revealed that the PD-pRBD group had a lower educational level and lowered BMI than the PD-NRBD group, which means that the educational level and BMI may have an impact on the occurrence of RBD.

The pathogenesis of PD is currently considered to be caused by the combined effects of age, genetics, and environmental factors. However, we did not identify any difference in smoking, alcohol consumption, tea-drinking, and pesticide exposure between the PD-pRBD and PD-NRBD groups, indicating that lifestyle and pesticide exposure had no significant effects on RBD.

The most obvious observation in this study was the higher prevalence of motor and non-motor features of PD in the PD-pRBD group than in the PD-NRBD group. The PD-pRBD group had been proved to have a longer disease duration, higher proportion of middle and late H-Y stage, and higher scores in both motor and non-motor scales, which is consistent with previous studies (30). The discussed results indicate that patients with PD-pRBD have more severe motor symptoms and worse conditions. However, in two other studies, patients in the PD-pRBD group were older and had longer disease duration than those in the PD-NRBD group, whereas the onset age was younger (31, 32), which might be due to the differences between the study population and the pRBD diagnostic tool.

This study found that the proportion of LEDD, dyskinesia, and wearing-off was higher in the PD-pRBD group than that in the PD-NRBD group, which was related to the increased dose of drugs in the PD-pRBD group. The frozen gait of the PD-pRBD group was also more common than that of the PD-NRBD group. A possible explanation for these results may be that the frozen gait was the middle-stage disability symptom of PD patients, and the proportion of PD-pRBD patients in the late-stage of PD was higher than that in the PD-NRBD group in this study. The pathogenesis of frozen gait is not well understood and may be associated with extensive neurological damage, including the brainstem (33) and pedunculopontine tegmental nucleus (PPN) (34). An autopsy of pathological study has shown that the loss of cholinergic neurons in the PPN of PD patients can result in freezing and falling, which may lead to a higher rate of frozen gait in the PD-pRBD group (35).

Several studies have shown that PD-pRBD patients are more likely to experience hallucinations than PD-NRBD patients (27). Many factors cause hallucinations in PD-pRBD patients, including the involvement of the PPN (34). A study using functional magnetic resonance has shown that weakened connections between the lateral geniculate body and the visual center of the cerebral cortex in patients with RBD can cause visual hallucinations (36). Studies have found that orthostatic hypotension is more common in PD-pRBD patients than in PD-NRBD patients (4). The pathogenesis of orthostatic hypotension is associated with sympathetic dysfunction. In the early stage of PD motor symptoms, the vagus dorsal nucleus would be damaged. The vagus nerve dorsal nucleus emits sympathetic nerve fibers to regulate the standing blood pressure. Sympathetic denervation during standing position cannot effectively cause peripheral vascular resistance to increase, which can lower the systolic blood pressure and lead to orthostatic hypotension (37).

It has been shown that RBD is a risk factor for cognitive dysfunction in patients with PD (4), especially in the field of attention and memory dysfunction (38). In a study of 32 patients with primary RBD, 22 patients with PD-RBD, 18 patients with PD-NRBD, and 40 healthy controls reported by Gagnon et al. (39), 50% of patients with RBD had mild cognitive impairment (MCI), 73% of PD-RBD patients had MCI, 11% of PD-NRBD patients had MCI, 8% of healthy controls had MCI, respectively, and the ratio of MCI in the PD-RBD group was much higher than that in the PD-NRBD group. Among the 2,440 patients with PD included in this study, MMSE was used to determine whether there was cognitive dysfunction; 15.9% of PD patients had cognitive dysfunction, 19.2% of PD-pRBD patients had cognitive dysfunction, and 13.6% of PD-NRBD patients had cognitive dysfunction, and the proportion of cognitive dysfunction in the PD-pRBD group was higher than that in the PD-NRBD group, which was consistent with the discussed literature. However, the prevalence of cognitive function in the PD patients included in this study was far below the earlier mentioned literature reports, which may be related to the population selection and sample size. The small sample size of Gagnon et al. may be one reason for this difference. To further study the clinical features of cognitive dysfunction in PD-pRBD patients and to explore the impact of pRBD on cognitive function in patients with PD, it is necessary to improve a variety of scores involving various cognitive domains, such as the Montreal Cognitive Assessment Scale, the Auditory Verbal Learning Test, the Boston Naming Test, and the Verbal Fluency Test.

This study found that the PD-pRBD group had lower PDSS scores than the PD-NRBD group, indicating that the total sleep quality of PD-pRBD patients was worse. pRBD can affect the quality of sleep at night, and studies have shown that PD-pRBD patients are more likely to have nighttime periodic leg movements, EDS, and nocturia than PD-NRBD patients (40), possibly leading to poor sleep quality in PD-pRBD patients. Previous studies have found that the incidence of sleep disorders in patients with PD is 40–90% (41–44), and the prevalence of EDS is approximately 20–60% (45, 46). The use of benzodiazepines or dopamine agonists, nocturnal sleep disorders, changes in the sleep/wake cycle; decreased orexin levels, autonomic dysfunction, and depression have been reported as risk factors for EDS (43, 47, 48). In our study, the prevalence of EDS was 32.4%, which is consistent with the discussed findings. Patients in the PD-pRBD group had a higher prevalence of EDS than the PD-NRBD group. A longer course of disease, severe disease, high LEDD, poor nighttime sleep quality, and severe orthostatic hypotension may be the risk factors for EDS.

In PD patients, constipation may occur before the appearance of RBD. The prevalence of constipation in patients with PD is 20–89% (49). In a multicenter study of 1,021 patients with PD, the prevalence of constipation was 85.4% (50). In our study, the prevalence of constipation was 48.2%, and the PD-pRBD group had a higher prevalence of constipation than the PD-NRBD group. The regression analysis showed that constipation was a risk factor for PD-pRBD. Olfactory dysfunction is another supporting criterion for the diagnosis of PD, and its prevalence in PD patients is 45–90% (51). In this study, the prevalence of olfactory dysfunction was 43.6%, which was slightly lower. Similar to the result of constipation, olfactory dysfunction was also more frequent in the PD-pRBD group and was identified as a risk factor for PD-pRBD. Analysis of depression also occurred in the same patient, which may be associated with longer disease duration, more severe symptoms, and more severe illness in PD-pRBD patients.

The study also found that the quality of life in the PD-pRBD group was worse than that in the PD-NRBD group. A possible explanation for this might be that PD-pRBD patients have more severe motor symptoms, more serious illness, and a higher proportion of other non-motor symptoms.

To date, few studies have focused on the difference between PD-prRBD and PD-poRBD. Also, we report some novel observations. This study found that 39.0% of PD-pRBD patients had pRBD prior to PD motor symptoms, and 61.0% of patients had pRBD after PD motor symptoms, indicating that 39.0% of patients with PD-pRBD develop from idiopathic pRBD, supporting the idea that pRBD is a PD predictor. The study of the relationship between pRBD and PD and the construction of the model requires a long-term follow-up cohort study of patients with idiopathic pRBD. Moreover, the severity of motor symptoms appeared worse in the PD-poRBD group.

The limitation of this study is that polysomnography is not used to diagnose RBD. However, the scale RBDQ-HK used in this study is simple and convenient and has high specificity and sensitivity for the evaluation and diagnosis of pRBD. Furthermore, the nature of a cross-sectional study was another limitation in our study. Therefore, longitudinal studies are essential to explore the clinical features and risk factors of RBD.

## Conclusion

In conclusion, we conducted a large sample study to systematically investigate the clinical features and associated factors of pRBD in a Chinese population of PD patients. We found that PD with pRBD presented with more severe motor and non-motor symptoms than PD without pRBD. Further, LOPD, long disease duration, high UPDRS-I score, high NMSS-4 score, low PDSS score, constipation, and hyposmia can be risk factors for pRBD in PD. Differences also occur between the PD-prRBD and PD-poRBD groups.

## Data Availability Statement

The original contributions presented in the study are included in the article/supplementary material, further inquiries can be directed to the corresponding author/s.

## Ethics Statement

The studies involving human participants were reviewed and approved by the institutional review board and ethics committee of Xiangya Hospital affiliated to Central South University. The patients/participants provided their written informed consent to participate in this study.

## Author Contributions

JG and BT conceived the study. KL, CW, YX, QX, QS, YT, and LF carried out the analysis and interpretation of the data. JL, ZW, and YY edited the manuscript. XY, BT, and JG discussed and revised the manuscript. All authors contributed to the article and approved the submitted version.

## Conflict of Interest

The authors declare that the research was conducted in the absence of any commercial or financial relationships that could be construed as a potential conflict of interest.

## References

[1] Postuma RB Gagnon JF Vendette M Montplaisir JY. Markers of neurodegeneration in idiopathic rapid eye movement sleep behaviour disorder and Parkinson's disease. Brain. (2009) 132(Pt 12):3298–307. 10.1093/brain/awp244 19843648

[2] Iranzo A Tolosa E Gelpi E Molinuevo JL Valldeoriola F Serradell M. Neurodegenerative disease status and post-mortem pathology in idiopathic rapid-eye-movement sleep behaviour disorder: an observational cohort study. Lancet Neurol. (2013) 12:443–53. 10.1016/S1474-4422(13)70056-5 23562390

[3] Postuma RB Iranzo A Hogl B Arnulf I Ferini-Strambi L Manni R. Risk factors for neurodegeneration in idiopathic rapid eye movement sleep behavior disorder: a multicenter study. Ann Neurol. (2015) 77:830–9. 10.1002/ana.24385 25767079 PMC5769479

[4] Schenck CH Boeve BF Mahowald MW. Delayed emergence of a parkinsonian disorder or dementia in 81% of older men initially diagnosed with idiopathic rapid eye movement sleep behavior disorder: a 16-year update on a previously reported series. Sleep Med. (2013) 14:744–8. 10.1016/j.sleep.2012.10.009 23347909

[5] Suzuki K Miyamoto M Miyamoto T Hirata K. Parkinson's disease and sleep/wake disturbances. Curr Neurol Neurosci Rep. (2015) 15:8. 10.1007/s11910-015-0525-5 25687697

[6] Postuma RB Berg D Stern M Poewe W Olanow CW Oertel W MDS clinical diagnostic criteria for Parkinson's disease. Mov Disord. (2015) 30:1591–1601. 10.1002/mds.26424 26474316

[7] Thenganatt MA Jankovic J. Parkinson disease subtypes. JAMA Neurol. (2014) 71:499–504. 10.1001/jamaneurol.2013.6233 24514863

[8] Lewis SJ Foltynie T Blackwell AD Robbins TW Owen AM Barker RA. Heterogeneity of Parkinson's disease in the early clinical stages using a data driven approach. J Neurol Neurosurg Psychiatry. (2005) 76:343–8. 10.1136/jnnp.2003.033530 15716523 PMC1739569

[9] Tomlinson CL Stowe R Patel S Rick C Gray R Clarke CE. Systematic review of levodopa dose equivalency reporting in Parkinson's disease. Mov Disord. (2010) 25:2649–53. 10.1002/mds.23429 21069833

[10] Shen SS Shen Y Xiong KP Chen J Mao CJ Huang JY. Validation study of REM sleep behavior disorder questionnaire-Hong Kong (RBDQ-HK) in east China. Sleep Med. (2014) 15:952–8. 10.1016/j.sleep.2014.03.020 24938584

[11] Chaudhuri KR Martinez-Martin P Brown RG Sethi K Stocchi F Odin P. The metric properties of a novel non-motor symptoms scale for Parkinson's disease: results from an international pilot study. Mov Disord. (2007) 22:1901–11. 10.1002/mds.21596 17674410

[12] Chaudhuri KR Pal S DiMarco A Whately-Smith C Bridgman K Mathew R. The Parkinson's disease sleep scale: a new instrument for assessing sleep and nocturnal disability in Parkinson's disease. J Neurol Neurosurg Psychiatry. (2002) 73:629–35. 10.1136/jnnp.73.6.629 12438461 PMC1757333

[13] Peng LL Li JR Sun JJ Li WY Sun YM Zhang R. [Reliability and validity of the simplified Chinese version of Epworth sleepiness scale]. Zhonghua Er Bi Yan Hou Tou Jing Wai Ke Za Zhi. (2011) 46:44–9. 10.1631/jzus.B1000197 21429336

[14] Aarsland D Litvan I Larsen JP. Neuropsychiatric symptoms of patients with progressive supranuclear palsy and Parkinson's disease. J Neuropsychiatry Clin Neurosci. (2001) 13:42–9. 10.1176/jnp.13.1.42 11207328

[15] Hamilton M. A rating scale for depression. J Neurol Neurosurg Psychiatry. (1960) 23:56–62. 10.1136/jnnp.23.1.56 14399272 PMC495331

[16] Montgomery SA Asberg M. A new depression scale designed to be sensitive to change. Br J Psychiatry. (1979) 134:382–9. 10.1192/bjp.134.4.382 444788

[17] Millar Vernetti P Perez Lloret S Rossi M Cerquetti D Merello M. Validation of a new scale to assess olfactory dysfunction in patients with Parkinson's disease. Parkinsonism Relat Disord. (2012) 18:358–61. 10.1016/j.parkreldis.2011.12.001 22227345

[18] Peto V Jenkinson C Fitzpatrick R Greenhall R. The development and validation of a short measure of functioning and well being for individuals with Parkinson's disease. Qual Life Res. (1995) 4:241–8. 10.1007/BF02260863 7613534

[19] Braak H Del Tredici K Rüb U De Vos RA Steur EN Braak E. Staging of brain pathology related to sporadic Parkinson's disease. Neurobiol Aging. (2003) 24:197–211. 10.1016/S0197-4580(02)00065-9 12498954

[20] Limousin N Dehais C Gout O Héran F Oudiette D Arnulf I. A brainstem inflammatory lesion causing REM sleep behavior disorder and sleepwalking (parasomnia overlap disorder). Sleep Med. (2009) 10:1059–62. 10.1016/j.sleep.2008.12.006 19345142

[21] Xi Z Luning W. REM sleep behavior disorder in a patient with pontine stroke. Sleep Med. (2009) 10:143–6. 10.1016/j.sleep.2007.12.002 18226960

[22] Winkelman JW James L. Serotonergic antidepressants are associated with REM sleep without atonia. Sleep. (2004) 27:317–21. 10.1093/sleep/27.2.317 15124729

[23] Iranzo A Santamaria J Tolosa E. The clinical and pathophysiological relevance of REM sleep behavior disorder in neurodegenerative diseases. Sleep Med Rev. (2009) 13:385–401. 10.1016/j.smrv.2008.11.003 19362028

[24] Gagnon JF Postuma RB Mazza S Doyon J Montplaisir J. Rapid-eye-movement sleep behaviour disorder and neurodegenerative diseases. Lancet Neurol. (2006) 5:424–32. 10.1016/S1474-4422(06)70441-0 16632313

[25] Rolinski M Szewczyk-Krolikowski K Tomlinson PR Nithi K Talbot K Ben-Shlomo Y. REM sleep behaviour disorder is associated with worse quality of life and other non-motor features in early Parkinson's disease. J Neurol Neurosurg Psychiatry. (2014) 85:560–6. 10.1136/jnnp-2013-306104 24187013 PMC3995329

[26] Mahlknecht P Iranzo A Högl B Frauscher B Müller C Santamaría J. Olfactory dysfunction predicts early transition to a Lewy body disease in idiopathic RBD. Neurology. (2015) 84:654–8. 10.1212/WNL.0000000000001265 25609758

[27] Gjerstad MD Boeve B Wentzel-Larsen T Aarsland D Larsen JP. Occurrence and clinical correlates of REM sleep behaviour disorder in patients with Parkinson's disease over time. J Neurol Neurosurg Psychiatry. (2008) 79:387–91. 10.1136/jnnp.2007.116830 17557796

[28] Yoritaka A Ohizumi H Tanaka S Hattori N. Parkinson's disease with and without REM sleep behaviour disorder: are there any clinical differences? Eur Neurol. (2009) 61:164–70. 10.1159/000189269 19129703

[29] Borek LL Kohn R Friedman JH. Phenomenology of dreams in Parkinson's disease. Mov Disord. (2007) 22:198–202. 10.1002/mds.21255 17133561

[30] Lee JE Kim KS Shin HW Sohn YH. Factors related to clinically probable REM sleep behavior disorder in Parkinson disease. Parkinsonism Relat Disord. (2010) 16:105–8. 10.1016/j.parkreldis.2009.08.005 19716333

[31] Poryazova R Oberholzer M Baumann CR Bassetti CL. REM sleep behavior disorder in Parkinson's disease: a questionnaire-based survey. J Clin Sleep Med. (2013) 9:55–9. 10.5664/jcsm.2340 23319905 PMC3525989

[32] Nihei Y Takahashi K Koto A Mihara B Morita Y Isozumi K. REM sleep behavior disorder in Japanese patients with Parkinson's disease: a multicenter study using the REM sleep behavior disorder screening questionnaire. J Neurol. (2012) 259:1606–12. 10.1007/s00415-011-6386-1 22231870

[33] Fasano A Herman T Tessitore A Strafella AP Bohnen NI. Neuroimaging of freezing of gait. J Parkinsons Dis. (2015) 5:241–54. 10.3233/JPD-150536 25757831 PMC4923721

[34] Hepp DH Ruiter AM Galis Y Voorn P Rozemuller AJ Berendse HW. Pedunculopontine cholinergic cell loss in hallucinating Parkinson disease patients but not in dementia with Lewy bodies patients. J Neuropathol Exp Neurol. (2013) 72:1162–70. 10.1097/NEN.0000000000000014 24226265

[35] Fling BW Cohen RG Mancini M Nutt JG Fair DA Horak FB. Asymmetric pedunculopontine network connectivity in parkinsonian patients with freezing of gait. Brain. (2013) 136(Pt 8):2405–18. 10.1093/brain/awt172 23824487 PMC3722352

[36] Geddes MR Tie Y Gabrieli JD McGinnis SM Golby AJ Whitfield-Gabrieli S. Altered functional connectivity in lesional peduncular hallucinosis with REM sleep behavior disorder. Cortex. (2016) 74:96–106. 10.1016/j.cortex.2015.10.015 26656284 PMC4820074

[37] Nakamura T Hirayama M Hara T Mizutani Y Suzuki J Watanabe H. Role of cardiac sympathetic nerves in preventing orthostatic hypotension in Parkinson's disease. Parkinsonism Relat Disord. (2014) 20:409–14. 10.1016/j.parkreldis.2014.01.003 24462345

[38] Chahine LM Xie SX Simuni T Tran B Postuma R Amara A. Longitudinal changes in cognition in early Parkinson's disease patients with REM sleep behavior disorder. Parkinsonism Relat Disord. (2016) 27:102–6. 10.1016/j.parkreldis.2016.03.006 27010070 PMC5031362

[39] Gagnon JF Vendette M Postuma RB Desjardins C Massicotte-Marquez J Panisset M. Mild cognitive impairment in rapid eye movement sleep behavior disorder and Parkinson's disease. Ann Neurol. (2009) 66:39–47. 10.1002/ana.21680 19670440

[40] Fantini ML Michaud M Gosselin N Lavigne G Montplaisir J. Periodic leg movements in REM sleep behavior disorder and related autonomic and EEG activation. Neurology. (2002) 59:1889–94. 10.1212/01.WNL.0000038348.94399.F6 12499479

[41] Lees AJ Blackburn NA Campbell VL. The nighttime problems of Parkinson's disease. Clin Neuropharmacol. (1988) 11:512–9. 10.1097/00002826-198812000-00004 3233589

[42] Sixel-Döring F Schweitzer M Mollenhauer B Trenkwalder C. Intraindividual variability of REM sleep behavior disorder in Parkinson's disease: a comparative assessment using a new REM sleep behavior disorder severity scale (RBDSS) for clinical routine. J Clin Sleep Med. (2011) 7:75–80. 10.5664/jcsm.28044 21344049 PMC3041623

[43] Kumar S Bhatia M Behari M. Sleep disorders in Parkinson's disease. Mov Disord. (2002) 17:775–81. 10.1002/mds.10167 12210875

[44] Tandberg E Larsen JP Karlsen K. A community-based study of sleep disorders in patients with Parkinson's disease. Mov Disord. (1998) 13:895–9. 10.1002/mds.870130606 9827612

[45] Setthawatcharawanich S Limapichat K Sathirapanya P Phabphal K. Validation of the Thai SCOPA-sleep scale for assessment of sleep and sleepiness in patients with Parkinson's disease. J Med Assoc Thai. (2011) 94:179–84. 10.1016/0045-6535(94)90113-9 21534364

[46] Videnovic A Noble C Reid KJ Peng J Turek FW Marconi A. Circadian melatonin rhythm and excessive daytime sleepiness in Parkinson disease. JAMA Neurol. (2014) 71:463–9. 10.1001/jamaneurol.2013.6239 24566763 PMC4078989

[47] Körner Y Meindorfner C Möller JC Stiasny-Kolster K Haja D Cassel W. Predictors of sudden onset of sleep in Parkinson's disease. Mov Disord. (2004) 19:1298–305. 10.1002/mds.20163 15389999

[48] Verbaan D van Rooden SM Visser M Marinus J van Hilten JJ. Nighttime sleep problems and daytime sleepiness in Parkinson's disease. Mov Disord. (2008) 23:35–41. 10.1002/mds.21727 17960797

[49] Pfeiffer RF. Gastrointestinal dysfunction in Parkinson's disease. Parkinsonism Relat Disord. (2011) 17:10–5. 10.1016/j.parkreldis.2010.08.003 20829091

[50] Maeda T Shimo Y Chiu SW Yamaguchi T Kashihara K Tsuboi Y. Clinical manifestations of nonmotor symptoms in 1021 Japanese Parkinson's disease patients from 35 medical centers. Parkinsonism Relat Disord. (2017) 38:54–60. 10.1016/j.parkreldis.2017.02.024 28279596

[51] Leentjens AF Parkinson disease: depression-risk factor or early symptom in Parkinson disease? Nat Rev Neurol. (2015) 11:432–3. 10.1038/nrneurol.2015.126 26215622

